# Fifty-six percent of proximal femoral cortical hypertrophies 6 to 10 years after Total hip arthroplasty with a short Cementless curved hip stem – a cause for concern?

**DOI:** 10.1186/s12891-019-2645-6

**Published:** 2019-05-29

**Authors:** Moritz M. Innmann, Johannes Weishorn, Thomas Bruckner, Marcus R. Streit, Tilman Walker, Tobias Gotterbarm, Christian Merle, Michael W. Maier

**Affiliations:** 1 Department of Orthopaedics and Trauma Surgery, Heidelberg University Hospital, Schlierbacher Landstrasse 200a, 69118 Heidelberg, Germany; 2 Institute of Medical Biometry and Informatics, University of Heidelberg, Im Neuenheimer Feld 130.3, 69120 Heidelberg, Germany; 3 Department of Orthopaedic and Trauma Surgery, Kepler University Hospital, Med Campus III Krankenhausstraße 9, 4021 Linz, Austria

**Keywords:** Cortical, Hypertrophy, Thigh, Pain, Short, Stem, Cementless, Hip, Arthroplasty

## Abstract

**Background:**

Thigh pain and cortical hypertrophies (CH) have been reported in the short term for specific short hip stem designs. The purpose of the study was to investigate 1) the differences in clinical outcome, thigh pain and stem survival for patients with and without CHs and 2) to identify patient and surgery-related factors being associated with the development of CHs.

**Methods:**

A consecutive series of 233 patients with 246 hips was included in the present retrospective diagnostic cohort study, who had received a total hip arthroplasty (THA) between December 2007 and 2009 with a cementless, curved, short hip stem (Fitmore, Zimmer, Warsaw, IN, USA). Clinical and radiographic follow-up, including the radiographic parameters for hip geometry reconstruction, were prospectively assessed 1, 3, and 6 to 10 years after surgery.

**Results:**

Cortical hypertrophies were observed in 56% of the hips after a mean of 7.7 years, compared to 53% after 3.3 years being mostly located in Gruen zone 3 and 5. There was no significant difference for the Harris Hip Score and UCLA score for patients with and without CHs. Only one patient with a mild CH in Gruen zone 5 and extensive heterotopic ossifications around the neck of the stem reported thigh pain. The Kaplan Meier survival rate after 8.6 years was 99.6% (95%-CI; 97.1–99.9%) for stem revision due to aseptic loosening and no association with CHs could be detected. Postoperative increase in hip offset was the only risk factor being associated with the development of CHs in the regression model (ΔHO; OR 1.1 (1.0–1.2); *p* = 0.001).

**Conclusions:**

The percentage of cortical hypertrophies remained almost constant in the mid-term compared to the short-term with the present cementless short hip stem design. The high percentage of cortical hypertrophies seems not be a cause for concern with this specific implant in the mid-term.

**Level of evidence:**

Diagnostic Level IV

## Background

Short hip stems show excellent survival rates ranging from 95 to 100% after 3 to 11 years, comparable to conventional cementless stems [1–6], but there is a lack of long-term data. Some disadvantages have been reported with short stems. Thigh pain occurred in 25% of patients after 2 to 4 years with the Tri-Lock Bone Preservation Stem (DePuy,Warsaw, IN,USA) and correlated with younger patient age, while only 2% of the hips showed cortical hypertrophy (CH) [7]. In contrast, a high rate of 29 to 63% for proximal femoral CHs was reported after 1.3 to 3.3 years with a cementless, curved, short stem (Fitmore, Zimmer, Warsaw, IN, USA), which is subject to the present investigation, while only 4% of the patients reported thigh pain [5, 8].

There is little evidence in the literature on factors contributing to the development of thigh pain and cortical hypertrophies and how they are associated [5, 7]. It has been hypothesized, both clinical findings might be related to the modulus mismatch between the distal part of the stem and the proximal femur affecting bone remodeling [7, 9]. This hypothesis is supported by a biomechanical study examining the mediolateral bending behavior of cementless stems, demonstrating a higher rigidity for a short compared standard tapered stem [9]. However, the definite relationship between thigh pain, cortical hypertrophies, load transmission and proximal implant loosening is still a matter of debate. As thigh pain and cortical hypertrophies may be of high clinical relevance with short hip stems, mid-term studies with adequate study cohort size are needed.

Therefore the present retrospective cohort study questioned

1. What are the differences in clinical outcome, thigh pain and stem survival for patients with and without CHs and
2. What patient and surgery-related factors are associated with the development of CHs?

## Methods

### Study cohort

The present retrospective diagnostic cohort study investigated a consecutive series of 233 patients with 246 cementless THAs from a single academic institution. The short-term results on the first 100 THAs have been previously published by the senior author in BMC Musculoskeletal Disorders reporting a percentage of 63% for proximal femoral CHs after 3.3 years [5]. In order to achieve a sufficient statistical power, all 233 patients were included in the study group, who had received a cementless THA between December 2007 and 2009 with a specific bone preserving curved stem (Fitmore stem, Zimmer, Warsaw, IN, USA), including the patients who had been followed in the previous short term study [5]. Indications for receiving the short stem were absence of severe proximal femoral deformity, adequate bone stock for cementless fixation and the diagnosis of primary osteoarthritis, developmental dysplasia of the hip (Crowe grade I), avascular necrosis of the femoral head, post-traumatic osteoarthritis, rheumatoid arthritis, slipped epiphysis of the femoral head or Perthes disease. The stem design for implantation was selected independently by each surgeon based on best endosteal press-fit fixation in the proximal femur and most accurate hip geometry reconstruction for hip offset and leg length difference according to templating [10]. Patients with bilateral THA or prior hip surgery were included in the study cohort. Diagnoses leading to THA and patient demographics are given in Table 1. In deceased patients, revision surgery between the last clinical follow-up and death was excluded using information from relatives, health insurance, general practitioners and clinical notes. Informed consent was obtained by all patients. The study was approved by the institutional review board (S—083/2017) and conducted according to the Helsinki Declaration of 2008.

**Table 1:** Demographics and diagnosis for patients with and without cortical hypertrophies

	Hips with CHs	Hips without CHs	*p*-value
Demographics			
Number of hips	105	83	
Gender (m: w)	57: 48	29: 44	0.32
Age at surgery in years	61 (53–68)	60 (51–68)	0.92
BMI (kg/m^2^)	26 (23–28)	26 (24–28)	0.46
HHS preoperatively	59 (43–67)	62 (48–69)	0.25
HHS postoperatively (3.3y FU)	97 (93–100)	97 (92–100)	0.89
HHS postoperatively (7.7y FU)	98 (93–100)	96 (91–100)	0.47
UCLA Score preoperatively	4 (3–6)	5 (3–6)	0.15
UCLA Score postoperatively (3.3y FU)	7 (6–7)	7 (6–8)	0.35
UCLA Score postoperatively (7.7y FU)	7 (5–7)	7 (5–7)	0.79
Diagnosis			
Primary osteoarthritis	61	46	0.71
Developmental dysplasia	22	28	0.05
Avascular necrosis	10	4	0.22
Posttraumatic osteoarthritis	4	1	0.27
Rheumatoid arthritis	5	4	0.93
Others	3	0	0.12

*CH* cortical hypertrophy, *FU* follow up; median values (interquartile range)

### Implants & Surgery

**Fig. 1:**
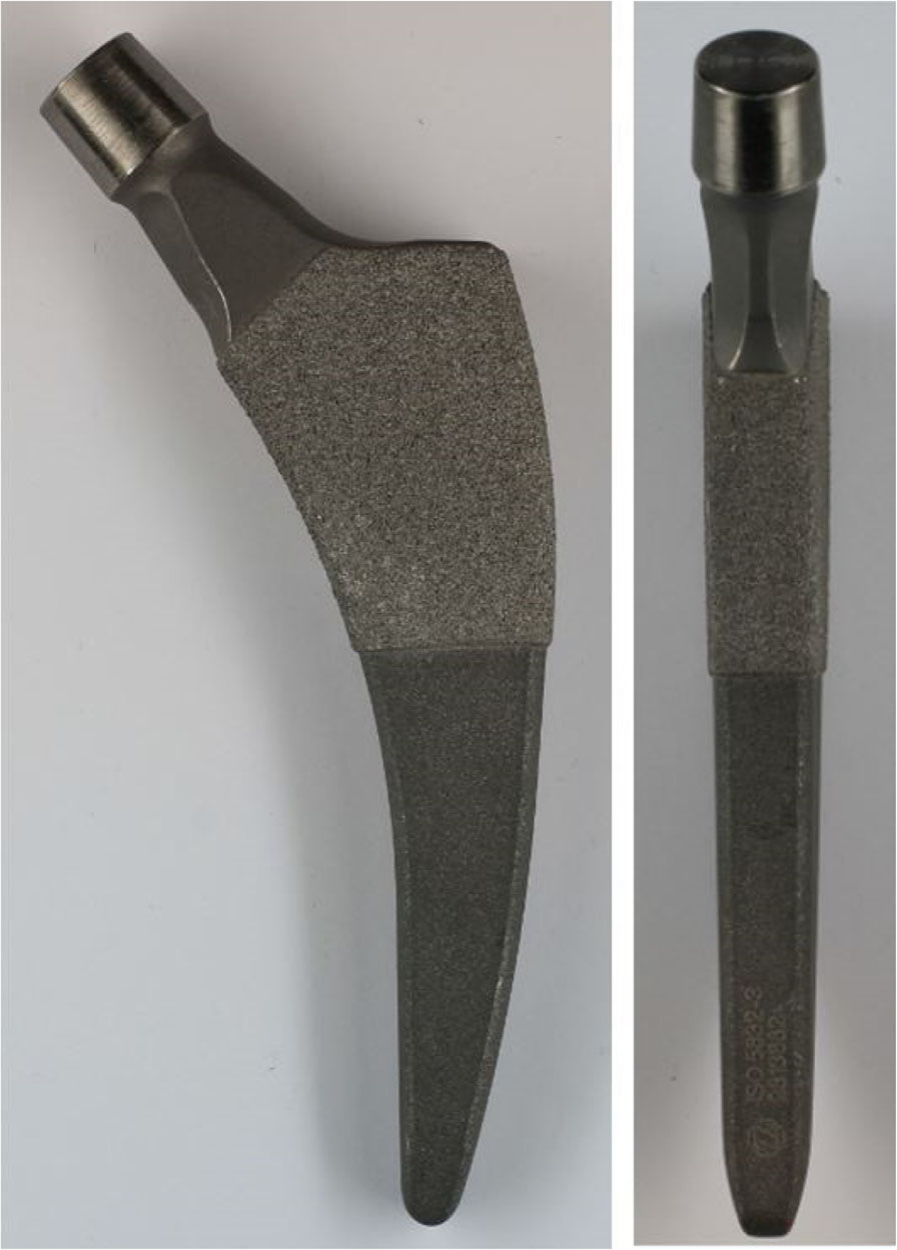
Photograph of the Fitmore® hip stem

A cementless, curved, short stem was used in all patients (Fitmore stem, Zimmer, Warsaw, IN, USA). The titanium alloy stem (Ti Al6V4) has a porolock Ti-VPS coating in the proximal part to enhance bone ingrowth and is available in four different neck angle options (127°, 129°, 137°, 140°) [8, 11]. The stem has a triple-tapered design to achieve press-fit fixation at the metaphyseal/diaphyseal level and according to the recommended femoral neck resection level, this stem can be classified as a trochanter-sparing or neck-harming short stem (Fig. 1) [5, 12, 13]. A cementless press-fit cup was used in 243 THAs (Allofit cup, Zimmer, Warsaw, IN, USA, 241 hips; Pinnacle cup, DePuy Synthes, West Chester, PA, USA, 2 hips). A reinforcement ring was used in two THAs due to one dysplastic and one posttraumatic acetabulum (Ganz reinforcement ring, Zimmer, Warsaw, IN, USA). A cemented cup was used in one THA due to osteopenia of the acetabular bone (Durasul, Zimmer, Warsaw, IN, USA).

Ceramic heads with three neck length options (− 4, 0, 4 mm; 28 mm or 32 mm diameter; Biolox forte®, CeramTec, Plochingen, Germany; 234 hips) or a CoCr femoral head with one neck length option (8 mm; 32 mm diameter; Metasul, Zimmer, Warsaw, IN, USA, 11 hips; Articul, DePuy Synthes, West Chester, PA, USA, one hip)) articulated with a highly cross-linked polyethylene liner (Durasul®; Zimmer, Warsaw, IN, USA). Hard on soft bearings with highly cross linked polyethylene liners were used in 244 THAs and ceramic on ceramic bearings were used in two THAs (Biolox, CeramTec, Plochingen, Germany).

Surgery was performed by five consultant surgeons in a university hospital setting using a modified, anterolateral Watson-Jones or a transgluteal Bauer approach. Intraoperative fluoroscopy was used in all patients with the final cup implant and the templated femoral broach in situ. Surgeons aimed for secure press-fit fixation, neutral stem alignment for varus/valgus position, anteversion of the stem of 10° ±10°, combined cup inclination/anteversion between 40 ± 10°/20 ± 10°, balanced leg length and reconstruction of the hip offset [14]. Standardized preoperative planning of the prosthesis size and position was performed in all patients.

### Clinical and radiographic assessment

Patients had clinical and radiographic follow-up examinations in regular intervals at 1, 3, and 5 to 10 years postoperatively. Preoperatively and at each time of follow-up, the Harris hip score (HHS) and the University of California, Los Angeles (UCLA) activity score were assessed [15, 16]. Standardized digital, calibrated AP hip, lateral hip and low-centred AP radiographs of the pelvis were acquired [17]. Radiographs were assessed as previously described for implant loosening, radiolucent lines, osteolysis, heterotopic ossification and cortical hypertrophies by two reviewers (MMI; JW), who were not involved in index surgery and blinded to each other [5]. Implant loosening was defined by the criteria of Engh et al. [18]. Osteolysis was defined as area with reduced bone stock or endostal resorption. Osteolysis, radiolucent lines and cortical hypertrophies were evaluated using the zones described by Gruen et al. [19]. Heterotopic ossifications were evaluated by the criteria of Brooker et al. [20]. The methods for radiographic measurements have been described previously in detail [10]. Briefly, varus/valgus stem alignment was measured as the difference in degrees between stem axis and proximal femoral shaft axis. The Canal Fill Index (CFI) was determined, to evaluate the meta−/ diaphyseal filling of the femoral canal by the cementless stem three centimeter below the lesser trochanter [21, 22]. Acetabular and femoral offset (AO, FO) were measured as the distance between the center of rotation of the femoral head and ipsilateral teardrop figure and the center of rotation and proximal femoral shaft axis, respectively. The postoperative offset change (ΔAO and ΔFO) was determined as the difference between pre- and postoperative AO and FO. Hip offset was defined as the sum of AO and FO [14]. Roman software V1.70 (Institute of Orthopedics, Oswestry, UK) and ImageJ software V1.44 (National Institute of Health, USA) were used for radiographic analysis.

**Fig. 2:**
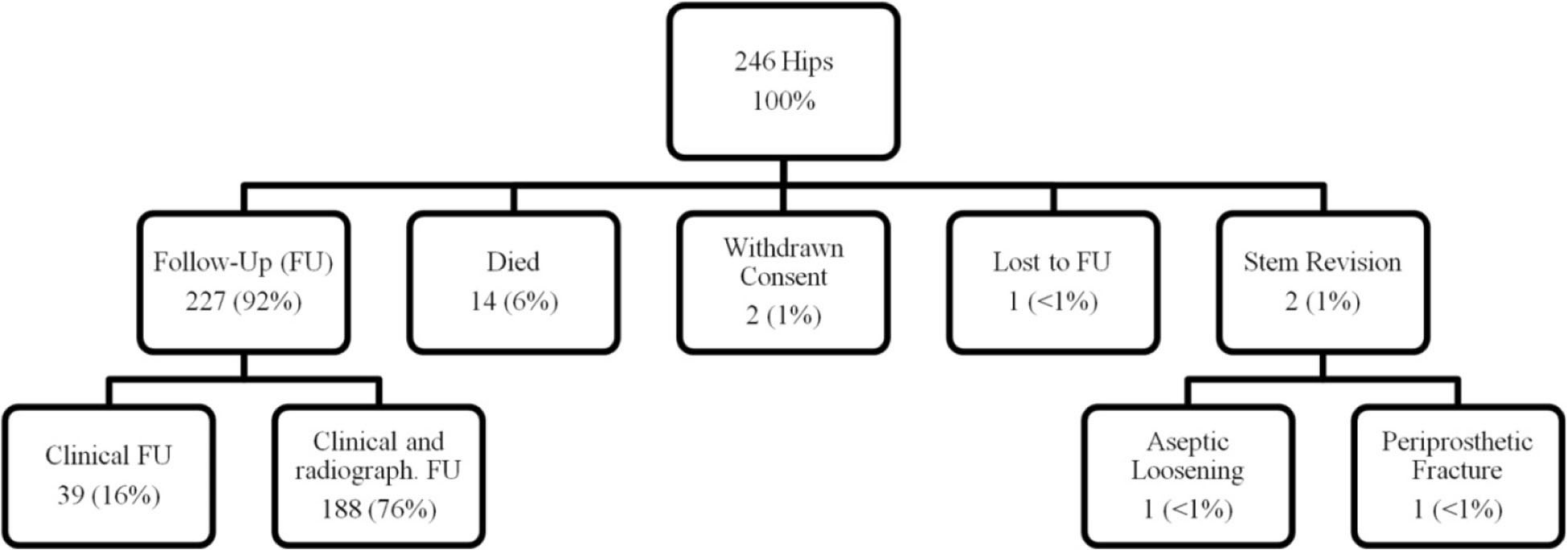
Distribution of hips at last Follow-Up (FU). Clinical follow up included data on patient reported outcome measures, thigh pain and stem survival. In patients with clinical and radiographic FU, additional up to date radiographs were available

At last follow-up, one patient of the study cohort was lost, 14 patients (14 hips, 6%) had died with the stem in situ, 2 patients (2 hips, 1%) had withdrawn their study consent and 2 patients (2 hips, 1%) had needed stem revision. Therefore, 214 patients (227 hips, 92%) were available for clinical follow-up after a mean of 7.7 years (6–10 years) with 188 patients having additional up to date radiographs (Fig. 2).

### Statistical analysis

After exploratory data analysis including Q-Q plots and Kolmogorov–Smirnov tests, non- parametric tests were used. Values are given as medians with interquartile range. To answer the first study question, group comparisons were performed using the Wilcoxon log-rank- or the Mann-Whitney-U-Test and stem survival was estimated using Kaplan-Meier survival analysis with 95% confidence intervals (CI). Patients were censored at death, at stem revision for different endpoints or at the end of follow-up, whichever came first. With regard to sample size, the survival was calculated to 8.6 years with a minimum of 17 hips still being followed up [23]. To answer the second study question, 171 hips could be included to calculate the odds ratios for potential risk factors being associated with the occurrence of a CH using a logistic regression model (in 11 patients with bilateral THAs, only the first hip was included and 6 patients had incomplete data sets, leaving 171 patients for analysis). A sample size calculation was performed according to the formula by Peduzzi et al. (*n* = 10 × k÷p) [24]. Based on the percentage (p) of 63% for CHs found in a previous study [5], and six predictors (k) being included in the regression model, a minimum of 95 hips would be needed to achieve sufficient power. We considered *p*-values of < 0.05 to be significant. SPSS® Version 24.0 (IBM SPSS Statistics, IBM, Armonk, NY, USA) and GraphPad Prism® Version 6.0 (GraphPad Software, San Diego, CA) were used to record and analyze the collected data. Intra- and interobserver reliabilities were calculated for 24 randomly selected data sets (10% of THAs), using average-measureintraclass-correlation coefficients (ICC) with a two-way random effects model for absolute agreement. Repeated measurements for intra- observer reliability were performed at day 1 and day 7 in a blinded fashion. The inter- and intra-observer correlation coefficients were excellent for radiographic measurements (range, 0.931 (95% CI; 0.813–0.972) to 0.997 (95% CI; 0.994–0.999).

## Results

**Fig. 3:**
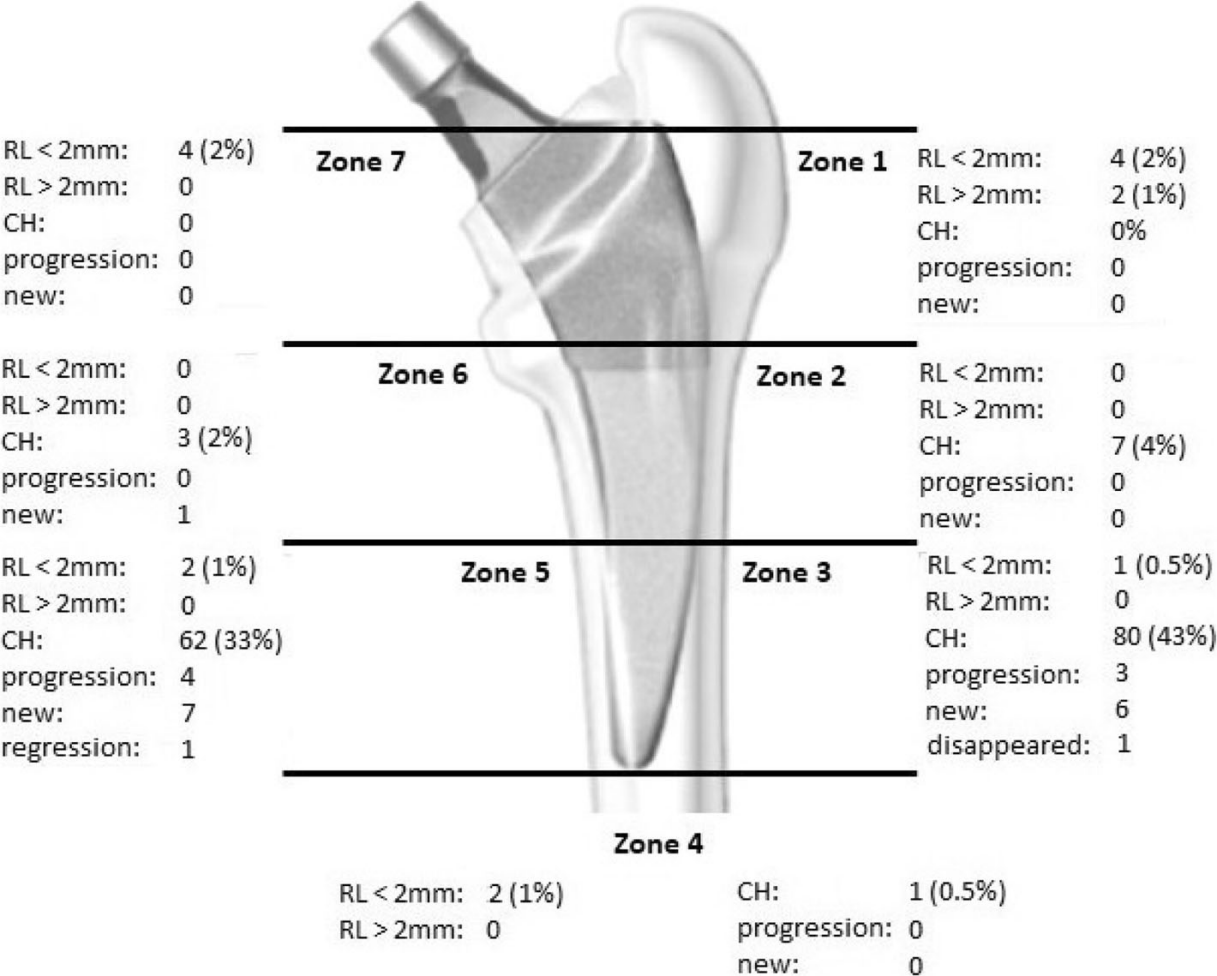
Distribution of radiolucencies (RL) and cortical hypertrophies (CH) around the 188 hip stems with available radiographic follow-up. Figure adapted according to Maier et al., 2015, BMC Musculoskeletal Disorders [5]

**Fig. 4:**
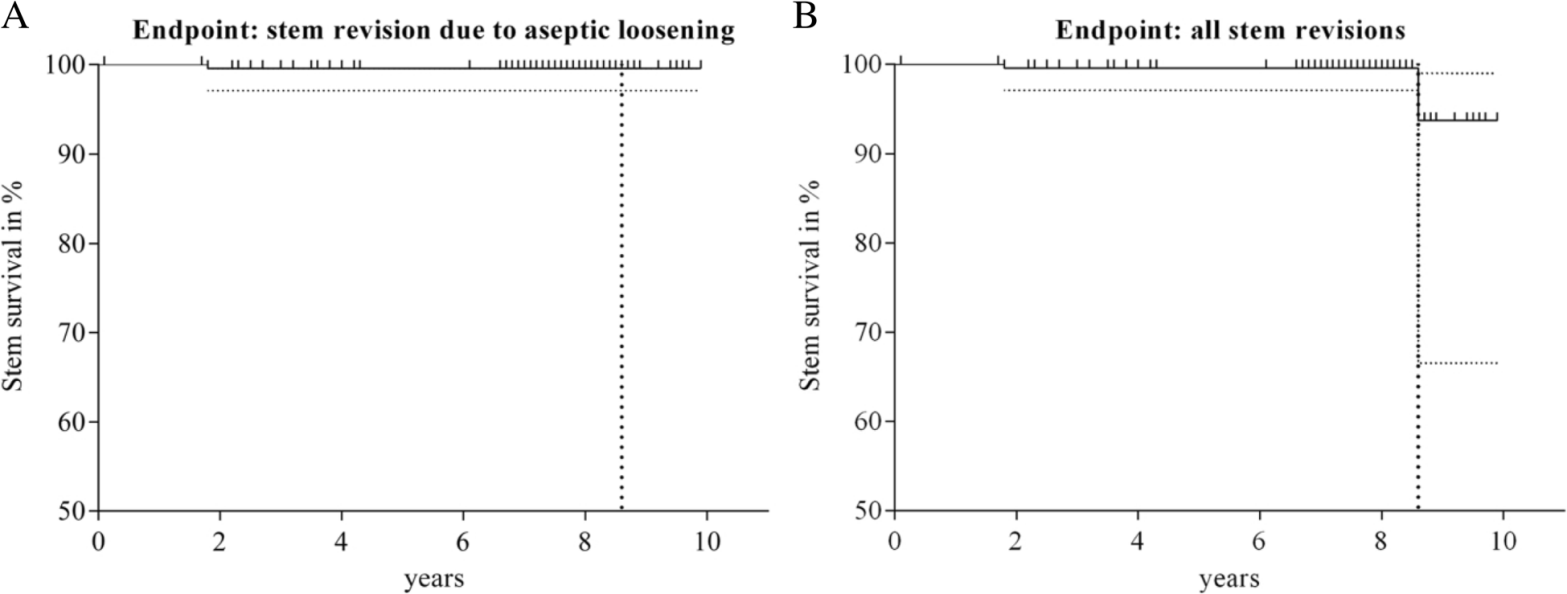
**a**&**b**: Kaplan Meier survival rate after 8.6 years for the endpoint A: "stem revision due to aseptic loosening (99.6%; 95%-CI; 97.1-99.9%) and for B: “all stem revisions” (93.7%; 95%-CI; 66.5–98.9%) (*n* = 246)

Cortical hypertrophies were observed in 56% of the hips (*n* = 105) and mostly distally located in Gruen zone 3 and 5, indicating a slight increase in the percentage of CHs compared to previous follow-up (53% after 3.3 years). The distribution and change of radiolucencies and hypertrophies are shown in Fig. 3. There was no significant difference for the HHS and UCLA score for patients with and without CHs (HHS: 98 (93–100) vs. 96 (91–100) points, *p* = 0.47 and UCLA: 7 (5–7) vs. 7 (5–7), (*p* = 0.79; (median (IQR)). One patient reported thigh pain. In this patient a mild CH was observed in Gruen zone 5 but extensive heterotopic ossification around the neck of the stem. No patient with a decrease in hip offset by more than 1 cm showed a limp and no patient with an increase in offset by more than 5 mm showed symptoms of a trochanteric bursitis. One stem revision was performed after 1.8 years for aseptic loosening due to continuous subsidence without primary fixation. A second stem revision was performed after a fall on the hip after 8.6 years for a periprosthetic Vancouver B2 fracture. The Kaplan Meier survival rate after 8.6 years was 99.6% (95%-CI; 97.1–99.9%) for the endpoint “stem revision due to aseptic loosening” and 93.7% (95%-CI, 66.5–98.9%) for the endpoint “all stem revisions” and no association with CHs and stem revision could be detected (Fig. 4 a&b).

**Fig. 5:**
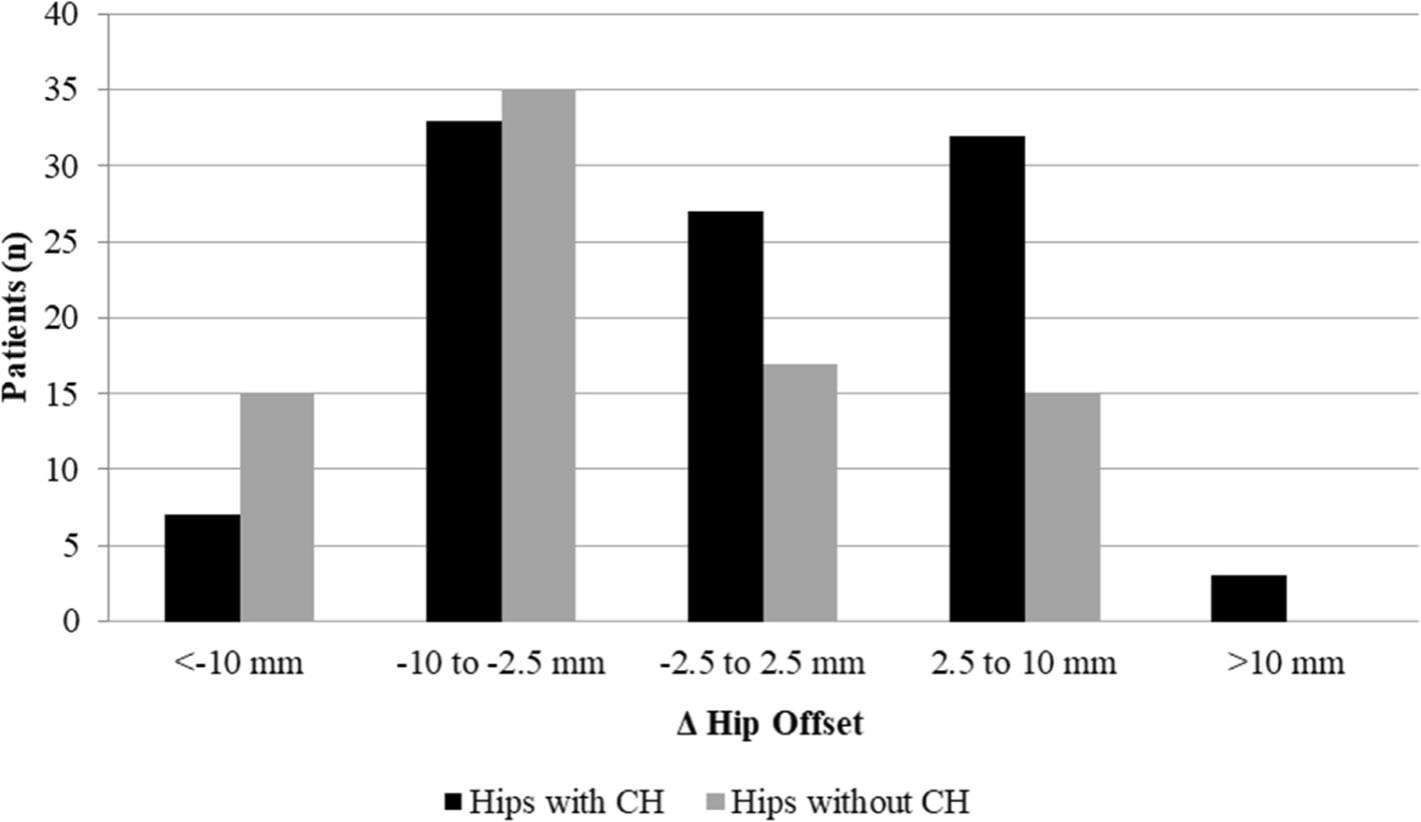
Histogram showing the distribution and proportion of hips with and without CHs depending on hip offset reconstruction (ΔHO). Patients with adequate or over-reconstructed hip offset demonstrated a higher proportion of hips with cortical hypertrophies (*n* = 188)

**Fig. 6:**
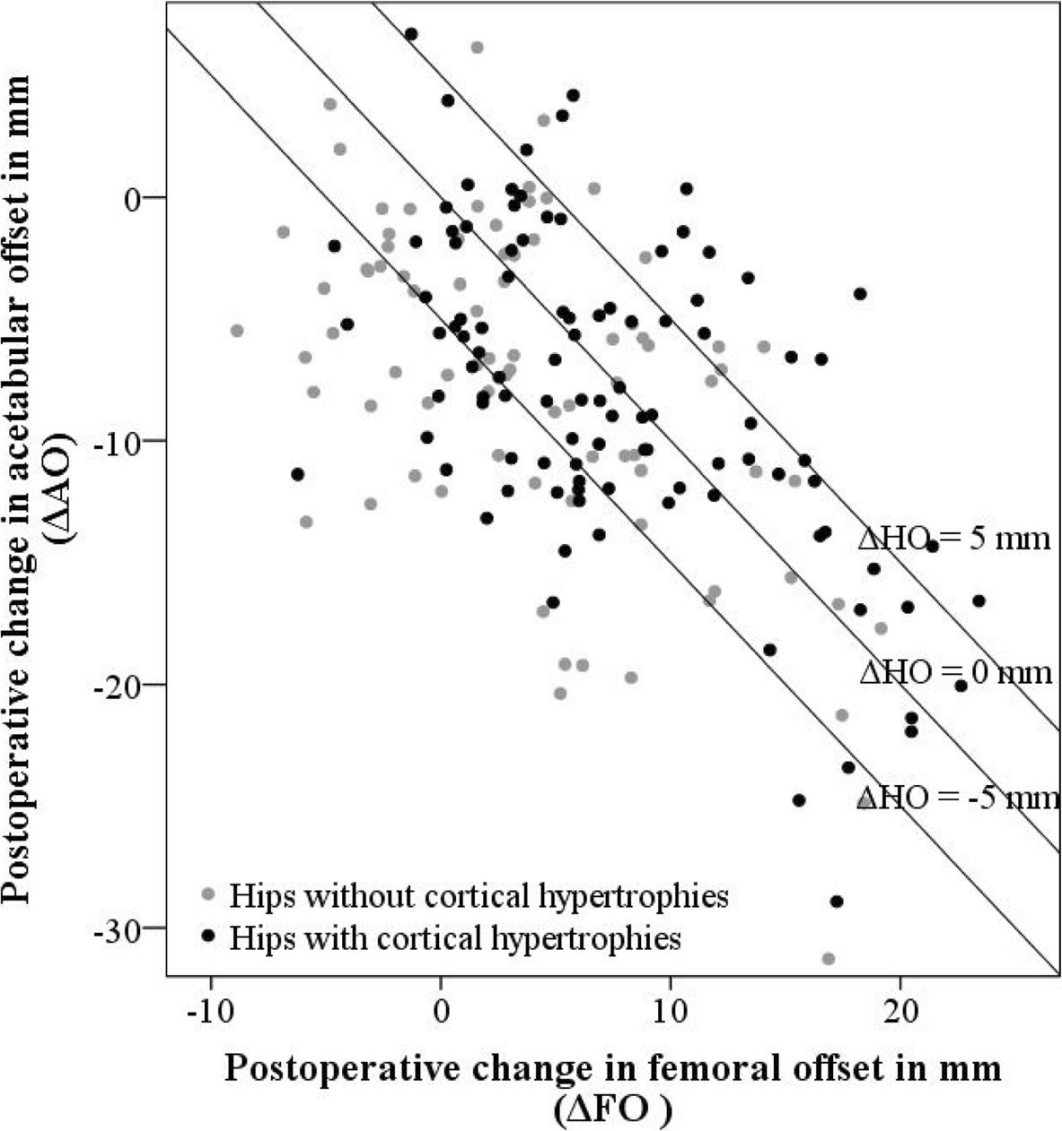
Scatter plot showing hips with (black) and without CHs (grey), dependent on change in hip offset reconstruction, with hip offset being the sum of femoral and acetabular offset (ΔHO = ΔFO + ΔAO) (*n* = 188)

An increase in hip offset was identified as the only risk factor for the development of CHs in the regression model (ΔHO; OR 1.1 (1.0–1.2); *p* = 0.001) (Table 2). Regarding effect size, median ΔHO was − 0.3 mm (− 7.7 – − 3.8) for hips with and − 3.8 mm (− 4.9–3.5) without CHs. The proportion of hips with CHs steadily increased with the increase in hip offset and only the two sub-groups with “under-reconstructed” hip offset (ΔHO < − 2.5 mm) showed a proportion of < 50% for hips with CHs (Figs. 5 and 6).

**Table 2:** Logistic regression model expressing the increased likelihood for the development of CHs dependent on hip offset reconstruction. (Nagelkerkes R^2^ = 0.118)

Model (*n* = 171)	Odds Ratio (95%-CI)	*p*-value
Stem axis	1.007 (0.881–1.151)	0.923
BMI	0.988 (0.921–1.059)	0.733
Age at surgery in years	0.989 (0.962–1.016)	0.405
Gender	0.608 (0.306–1.21)	0.157
Canal Fill Index	1.817 (0.092–36.007)	0.581
Δ Hip Offset in mm	1.104 (1.044–1.168)	0.001*

*indicating significance (*p* < 0.05)

## Discussion

The concept of short femoral stems was recently described as being attractive, but concerns were expressed due to a lack of data in the literature and more structured and specific research was recommended [25]. It was hypothesized that in the future, short stems may become the norm, but we were a long way from that [25]. In the face of this current trend, the present findings regarding clinical outcome, thigh pain, stem survival and cortical hypertrophies are of clinical relevance.

To our knowledge, this is the first study reporting a constant high percentage of 56% of cortical hypertrophies for this specific cementless curved, short stem design in the mid-term after 6 to 10 years compared to the short term after 2 to 4 years. Despite the present radiographic findings, there was no difference in clinical outcome and stem survival for patients with and without CHs and the only patient or surgery-related factor being associated with the development of CHs was reconstruction of hip offset. Surgeons aimed to reconstruct hip offset in order to achieve optimal clinical outcome, which has been recently demonstrated to correlate with accurate HO reconstruction and minimized leg-length difference [14]. Under-reconstruction of hip offset should be avoided as it is associated with inferior clinical outcome and increased risk for dislocation, as soft tissue tension is reduced, while potential negative effects of a slight increase in hip offset are still controversially discussed [14, 26]. In the present study, subgroups with adequately restored to increased hip offset (− 2.5 mm to > + 10 mm) demonstrated percentages of more than 50% for hips with CHs. In order to achieve optimal outcome with this short stem and reconstructing hip offset, the increased likelihood of developing CHs might have to be accepted. As no relevant number of radiolucencies or osteolysis could be observed in the proximal Gruen zones and stem survival was excellent in the mid-term, we suggest interpreting the high percentage of CHs as bone remodeling due to a potential distal load transfer but not as a sign of proximal stem loosening.

Interpreting our results in context of the literature, the large variety of short hip stems and different classifications have to be acknowledged [12, 13]. Applying the classifications from the literature, the Fitmore stem can be characterized as a trochanter-sparing or neck-harming cementless stem [12, 13]. Due to the variety of “short”-stem designs with individual patterns of cementless fixation and load transfer, the comparison of different stem designs regarding bone remodeling and occurrence of cortical hypertrophies is difficult. A systematic review and meta-analysis by Yan et al. reported similar periprosthetic bone remodeling around several short- and standard stems, with moderate quality evidence [27]. In particular, bone remodeling of the Fitmore stem compared to the CLS stem demonstrated significantly less bone mineral density reduction in the distal, lateral femur, equivalent to Gruen zones 2&3 in a prospective randomized study [28]. This finding supports our assumption, that the present distal femoral cortical hypertrophies might be attributable to bone remodeling with pronounced distal load transfer. However, surgeons should be aware that asymptomatic CHs should be distinguished from symptomatic CHs presenting with thigh pain because stress fractures around a short cementless stem without trauma have been reported in rare cases [29]. Stem survival was good in the present study with 93.7% after 8.6 years for the endpoint all stem revisions comparing well to other trochanter sparing stem designs with survival rates of 90 to 98% after 7 years [12]. Furthermore, no trend towards an increased revision rate for stems with CHs could be observed in our study while the rate of CHs remained almost constant at 56% after 7.7 years compared to 53% after 3.3 years. With respect to clinical outcome, no difference could be demonstrated for the HHS and UCLA score for patients with and without CHs in the mid-term while thigh pain was observed in only one patient.

The study has limitations that have to be addressed. During the study period 246 of 836 THAs were performed with the studied short stem and 506 with a cementless tapered straight stem. The reviewed hip stem was chosen by surgeons over the standard tapered straight stem, when offering a more precise option of press-fit fixation and hip anatomy reconstruction during templating. Therefore, a potential preoperative selection bias cannot be excluded due to the retrospective study design, but we tried to minimize this bias by including all patients with the short stem during the study period. A further limitation due to the retrospective study design is that no bone mineral density measurements were performed preoperatively and consecutively thereafter. Nevertheless, the percentage of 76% for clinical and radiological follow-up was high and only one patient (0.5%) lost. In combination with the excellent ICCs, indicating high reliability of the radiographic measurements, the present study was adequately designed and powered to answer our study questions. Although, the regression analysis demonstrated that hip offset restoration was the only risk factor being associated with the development of CHs, the explanatory power of the model and clinical relevance of hip offset restoration for the occurrence of cortical hypertrohies has to be interpreted with caution due to the small R^2^-value (0.118) and the wide 95%-CI for the canal fill index. Therefore, the model might be underpowered for the factor canal fill index. Lastly, stem design results in an individual fixation pattern and load transmission to the proximal femur. Therefore, care must be taken when applying the presented findings on cortical hypertrophies and thigh pain to different stem designs.

## Conclusion

Excellent clinical outcome, without differences for thigh pain and stem survival was observed for patients with and without CHs. The only patient and surgery-related factor being associated with the development of CHs was the change in hip offset. Therefore, we assume that the high percentage of cortical hypertrophies is not a cause for concern with this specific stem design in the midterm, but long term follow-up is needed to confirm this assumption.

## Data Availability

The dataset used and analysed in the current study is available from the corresponding author on reasonable request.

## Abbreviations

AO: Acetabular offset
CFI: Canal Fill Index
CH: Cortical hypertrophy
CI: Confidence intervals
Fig.: Figure
FO: Femoral offset
HHS: Harris Hip Score
HO: Hip offset
ICC: Intraclass-correlation coefficients
IQR: Interquartile range
OR: Odds ratio
THA: Total hip arthroplasty
UCLA: University of California, Los Angeles
ΔAO: Postoperative change in acetabular offset compared to preoperatively
ΔFO: Postoperative change in femoral offset compared to preoperatively
ΔHO: Postoperative change in hip offset compared to preoperatively
